# Ammonium is a key determinant on the dietary restriction of yeast chronological aging in culture medium

**DOI:** 10.18632/oncotarget.2989

**Published:** 2014-12-03

**Authors:** Júlia Santos, Fernanda Leitão-Correia, Maria João Sousa, Cecília Leão

**Affiliations:** ^1^ Life and Health Sciences Research Institute (ICVS), School of Health Sciences, University of Minho, Braga, Portugal; ^2^ ICVS/3B's - PT Government Associate Laboratory, Braga, Guimarães, Portugal; ^3^ Molecular and Environmental Biology Centre (CBMA), Department of Biology, University of Minho, Braga, Portugal

**Keywords:** Ammonium, lifespan, dietary restriction, amino acid restriction, yeast

## Abstract

New evidences have recently emerged from studies in yeast and in higher eukaryotes showing the importance of nutrient balance in dietary regimes and its effects on longevity regulation. We have previously shown that manipulation ofammoniumconcentration in the culture and/or aging medium can drastically affect chronological lifespan (CLS) of *Saccharomyces cerevisiae*, especially in amino acid restricted cells. Here we describe that the CLS shortening under amino acid restriction can be completely reverted by removing ammonium from the culture medium. Furthermore, the absence of ammonium, and of any rich nitrogen source, was so effective in extending CLS that no beneficial effect could be observed by further imposing calorie restriction conditions. When present in the culture medium,ammoniumimpaired the consumption of theauxotrophy-complementing amino acidsand caused in an improper cell cycle arrest of the culture. *TOR1* deletion reverted ammonium effects both in amino acid restricted and non-restricted cultures, whereas, Ras2p and Sch9p seem to have only a milder effect in the mediation ofammonium toxicity under amino acid restriction and no effect on non-restricted cultures. Our studies highlight ammonium as a key effector in the nutritional equilibrium between rich and essential nitrogen sources and glucose required for longevity promotion.

## INTRODUCTION

The budding yeast *Saccharomyces cerevisiae* is a highly exploited model to study environmental and genetic factors affecting longevity. The aging process is conserved from yeasts to mammals, with several studies showing that reducing growth factors/nutrients intake has profound positive effects in extension of life span and also improves overall health by delaying or reducing aged-related diseases in mammals [1]. The manipulation of well conserved nutrient-signaling pathways can be accomplished by dietary restriction (DR), in which the intake of nutrients, and not necessarily calories, is reduced without causing malnutrition, or by caloric restriction (CR) usually referring to a 20-40% reduction in calorie intake [1]. In yeast, the vast majority of protocols for CR are based on the decrease of glucose concentration in the medium from the standard 2% to 0.5 or 0.05%. [2, 3]. Studies using CR show that reducing glucose concentration in culture media is sufficient to increase replicative and chronological life span (CLS) [4-8]. Further studies have revealed that the major nutrient-signaling pathways TOR, SCH9 and RAS/AC/PKA are involved in longevity regulation by glucose, promoting cell division and growth in response to nutrients while inhibiting the general stress response and autophagy [6, 9].

Manipulation of several single components of the culture medium is known to extend CLS in yeast [2, 3, 10-12] and so in the past couple of years many medium components have surfaced as aging affecting factors [13-15]. Components of the culture media other than glucose, such as amino acids [3, 10-12, 16] and factors like the products of fermentation have also been implicated in the regulation of CLS [17, 18]. Acidification of the culture media mainly due to acetic acid and other organic acids production negatively impacts CLS [18]. Ethanol is another fermentative metabolite capable of inducing CLS reduction in aged cells by yet unknown mechanisms [17, 19]. These products of fermentation are believed to act as carbon sources and activate pro-aging pathways, thus preventing cells from entering a CR state [13]. A recent study postulates that not only individual components of the medium but rather a nutritional balance of medium composition, greatly affects yeast longevity [14]. The authors showed that a balance between glucose, amino acids and yeast nitrogen base (YNB), played a significant role in regulation of yeast CLS. The well-known lengthening effect of CR on longevity seems to be dependent on other nutrients in the medium, instead of glucose alone. It further suggests that nutrient composition is an important factor for longevity of budding yeast and the three nutrients and their interactions play different roles in the lifespan of different strains [14]. Furthermore, new evidences have emerged linking DR to anti-aging effects by elucidating how glucose and amino acids threonine, valine and serine modulate stress and aging in yeast cells [20].

Another pro-aging factor recently implicated on yeast CLS is DNA replication stress that culminates in a cell failure to arrest in G0/G1 phase, leading to replication stress-induced genome instability and apoptosis [21]. DNA replication stress has also been reported as a major cause of genome instability at early stages of cancer [22]. Also a correlation between CLS extension and a more efficient G0/G1 arrest was found in mutational inactivation of conserved RAS, TOR and SCH9 nutrient-signaling pathways or in calorie restriction conditions [23, 24]. Other studies additionally revealed that starvation of “natural” nutrients such as phosphate and sulphate leads to an arrest in G0/G1 cell cycle phase of prototrophic cells, while auxotrophic cells failed to arrest the cell cycle upon starvation of essential nutrients (auxotrophic nutrients) [25]. These findings clearly reveal a failure of auxotrophic cells in regulating nutrient sensing in response to starvation of essential nutrients [16]. Furthermore, limiting levels of auxotrophy-complementing amino acids, in the growth medium, induced an early arrest in G2/M phase, negatively affecting chronological longevity and leading to a premature aging phenotype [11].

In yeast, not all nitrogen sources are equally preferred, being selected through the nitrogen catabolite repression (NCR) mechanism also known as nitrogen discrimination pathway (NDP) [26]. This pathway enables yeast to repress genes that code for proteins required for the use of poor nitrogen sources, when in the presence of sufficient quantities of rich nitrogen sources like glutamine [27]. Another major transcriptional regulatory system in nitrogen metabolism is the general amino acid control pathway (GAAC). The GAAC was first described as a stress response pathway that, in reaction to amino acid starvation, activates the protein kinase Gcn2p (general amino acid control non-derepressible 2) to phosphorylate the eukaryotic initiation factor-2 (eIF2p) reducing its activity and thus lowering global translation while at the same time, preferentially stimulating translation of *GCN4* mRNA [27, 28].

In yeast as well as in higher eukaryotes, cells sense amino acid levels by two conserved signal-transducing kinases, the eIF2*α* kinase GCN2 and TOR kinase [29]. Cells are capable of sensing not only absence of individual amino acids but also the presence of more adequate ones. In response to single amino acid depletion, uncharged tRNAs accumulate and activate Gcn2p [30]. On the contrary, TOR kinase seems to respond to the presence, rather than the absence, of specific amino acids. Regulation of both GCN2 and TOR kinases in amino acid sensing has been described as having a major impact on dietary restriction longevity [29].

Our previous studies determined a role for ammonium (NH_4_^+^), a good nitrogen source commonly used by *S. cerevisiae*, as an extrinsic factor negatively affecting the yeast longevity, the effect being positively correlated with the concentration of NH_4_^+^ added to the culture medium [31]. We further demonstrated that NH_4_^+^ toxicity during yeast aging in water depends on the specific auxotrophy-complementing amino acid they are deprived of, and provided new insights in the modulation of CLS by NH_4_^+^, linking NH_4_^+^ toxicity to amino acid limitation [32].

Herewith, our findings support the view that the CLS shortening observed under auxotrophy-complementing amino acids restriction can be reverted by removing the non-limiting good nitrogen sources (NH_4_^+^ or glutamine in the present work) from the culture medium and that the abundance of these non-limiting good nitrogen sources also affects yeast CLS preventing its expansion. Shortening of CLS in these conditions was accompanied by the induction of replicative stress and by an impairment of the essential amino acids consumption. We further demonstrate that NH_4_^+^ is a necessary nutrient for the beneficial effects of caloric restriction on longevity to occur, highlighting the importance of a nutritional equilibrium between several nutrients, other than glucose alone, on longevity regulation.

## RESULTS

### The CLS shortening induced under auxotrophy-complementing amino acids restriction can be reverted by removing ammonium or another non-limiting good nitrogen source from the culture medium

**Figure 1:**
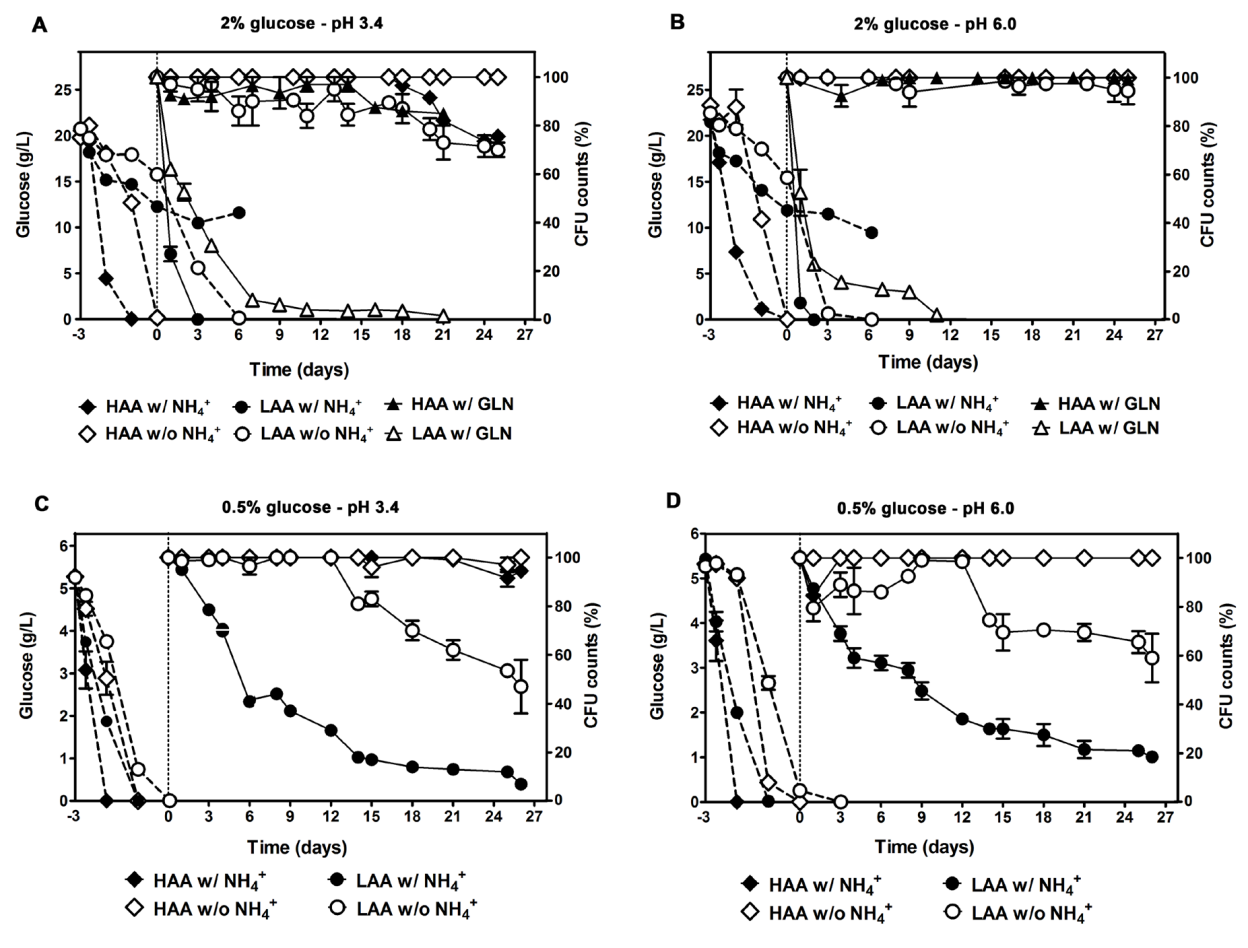
CLS shortening under amino acid restriction is reverted by removing the non-limiting nitrogen source (ammonium or glutamine) from the culture medium and no beneficial effect could be observed by further imposing caloric restriction Glucose consumption (dashed lines) and survival (CFU counts, %; solid lines) of *S. cerevisiae* BY4742 cultured in SD media buffered to: pH 3.4 (A and C) or pH 6.0 (B and D), with 2% (A and B) or 0.5% (C and D) glucose and supplemented with: high and low concentrations of auxotrophy-complementing amino acids (HAA and LAA, respectively), and with (w/) or without (w/o) NH_4_^+^ [05%, (NH_4_)_2_SO_4_] or glutamine [700 mg/L, GLN] supplementation. In all the cultures, starting cell density was about 3.8 × 10^7^ cells/ml. Day -3 represents the day of culture inoculation and day zero represents the beginning of aging experiments. Values are means ± SEM (n=3).

We have previously established that NH_4_^+^ at the concentration commonly used in SD medium [0.5%, (NH_4_)_2_SO_4_], is toxic for aging yeast particularly when grown under amino acid restriction conditions, leading to cell death in a concentration dependent manner, with a significant increase in cell survival being observed when the NH_4_^+^ concentration in the medium was decreased to 0.01% [31]. We now pursued to investigate how the CLS is modulated by the complete absence of NH_4_^+^, with or without auxotrophy-complementing amino acids restriction, and further evaluated the specificity of the NH_4_^+^ effect on the CLS by extending the studies to glutamine, another good nitrogen source for the yeast *S. cerevisiae*. Results presented in Fig. 1A and 1B, showed that in cultures with amino acid restriction (low concentrations of auxotrophy-complementing amino acids – LAA medium) and without NH_4_^+^ supplementation, cells did not lose their viability during 25 days of CLS experiments, maintaining it close to maximum value (100% CFU). On the contrary, in the same media but in the presence of NH_4_^+^ a rapid shortening of CLS was observed, with no viable cells being detected after 3 days. Furthermore, cells aged in the first condition (LAA medium, without NH_4_^+^ supplementation) displayed a CLS that was almost identical to the ones grown without amino acids restriction (high concentrations of auxotrophy-complementing amino acids – HAA medium), either in the presence or absence of NH_4_^+^ (Fig. 1A and 1B).

In order to evaluate the specificity of the NH_4_^+^ effect on the CLS shortening we replaced in the culture medium NH_4_^+^ by glutamine. The results indicated that, like for NH_4_^+^, cells grown in LAA medium with glutamine lost viability fast displaying a very short CLS, as compared with that of cells grown in LAA medium in the absence of a good nitrogen source (Fig. 1A and 1B). Also and importantly, for LAA medium, glutamine supplementation resulted in a CLS shortening that was similar to the one of LAA cells with NH_4_^+^ in the medium, suggesting that the shortening effect in CLS is not specific of NH_4_^+^, rather it appears that it may be induced by other non-limiting good nitrogen sources like glutamine. Thus, these results together strongly suggest that the CLS shortening that takes place in SD medium with 2% glucose under essential amino acids restriction is mainly due to the presence of non-limiting good nitrogen sources in the culture medium (NH_4_^+^ or glutamine, in the present work) since in their absence no significant difference in CLS was observed between cells cultured in HAA or LAA media. Therefore, the CLS shortening effect described in SD medium with 0.5% of NH_4_^+^ sulphate and 2% glucose as being induced by amino acid restriction [11] can be reverted by removing the non-limiting good nitrogen sources from the culture medium. These conclusions are valid both for buffered (Fig. 1) and non-buffered SD medium (Fig. S1).

### The CLS extending effect of caloric restriction attained by decreasing glucose concentration is not observed in the absence of a non-limiting good nitrogen source

As it was referred in introduction, the nutritional balance of medium composition, greatly affects yeast longevity and the effect of caloric restriction on longevity may be dependent on other nutrients in the medium, instead of glucose alone [14]. Herewith, we tested if this nutritional balance requirement is also applied to NH_4_^+^ and therefore, if its effects on CLS are influenced by differences in glucose concentrations in combination with restriction or not of essential amino acids concentrations. We started by measuring glucose consumption in media with 2% glucose under the conditions used in previous section. As it is shown by the results presented in Fig. 1A and Fig. 1B the sugar was totally depleted in media with high amino acid concentrations (HAA medium), with or without NH_4_^+^, although a delay of one day was encountered in the latter medium. On the other hand, glucose consumption was much slower in medium with low amino acid concentrations (LAA medium), being depleted only after 6 days in medium lacking NH_4_^+^, and not being completely consumed for medium with NH_4_^+^ wherein a fast loss of cell viability occurred. Next, we measured CLS in the same media, but imposing caloric restriction by decreasing glucose concentration from 2% to 0.5%. For all the conditions tested (LAA or HAA media supplemented with 0.5% glucose, with and without NH_4_^+^, pH 3.4 and 6.0) glucose was completely depleted before the beginning of the aging period, day 0 (Fig. 1C and 1D). Regarding the CLS pattern, the results showed that for the HAA medium without NH_4_^+^, during the experimental period of 21-26 days no significant differences were observed in the CLS measured either in 2% or 0.5% glucose at both pH values. In HAA medium with NH_4_^+^, for 0.5% glucose, comparatively with 2% glucose medium, a slight extension in the CLS started to be observed after day 18 (Fig. 1C) which is in agreement with expected caloric restriction effect. As to LAA medium with NH_4_^+^, decreasing the glucose concentration, from 2% to 0.5%, strongly increased the CLS, cell viability being still measurable at day 25 in 0.5% glucose medium, whereas for 2% glucose no viable cells could be detected after day 3. On the other hand, for LAA medium without NH_4_^+^, no extension of CLS was observed for cells grown in 0.5% when compared with 2% glucose and, on the contrary, the imposed caloric restriction was associated with a CLS shortening (Fig. 1).

It should be highlighted that in LAA medium with 0.5% glucose, the presence of NH_4_^+^ still induces a much faster decrease in cell viability than in its absence, suggesting that, independently of caloric restriction, NH_4_^+^ appears to be the major responsible for the observed CLS shortening under amino acid restriction.

In short, data suggest that the caloric restriction effect can be observed under amino acid restriction conditions in the presence of NH_4_^+^, but not in its absence. Therefore, this indicates that NH_4_^+^ mediates the required nutritional signaling that allows the beneficial effect of caloric restriction on longevity to occur, as described for other set of nutrients [14].

### The majority of ammonium during aging is not consumed and its presence in the medium is associated with a decrease of the consumption of the auxotrophy-complementing amino acids

**Figure 2:**
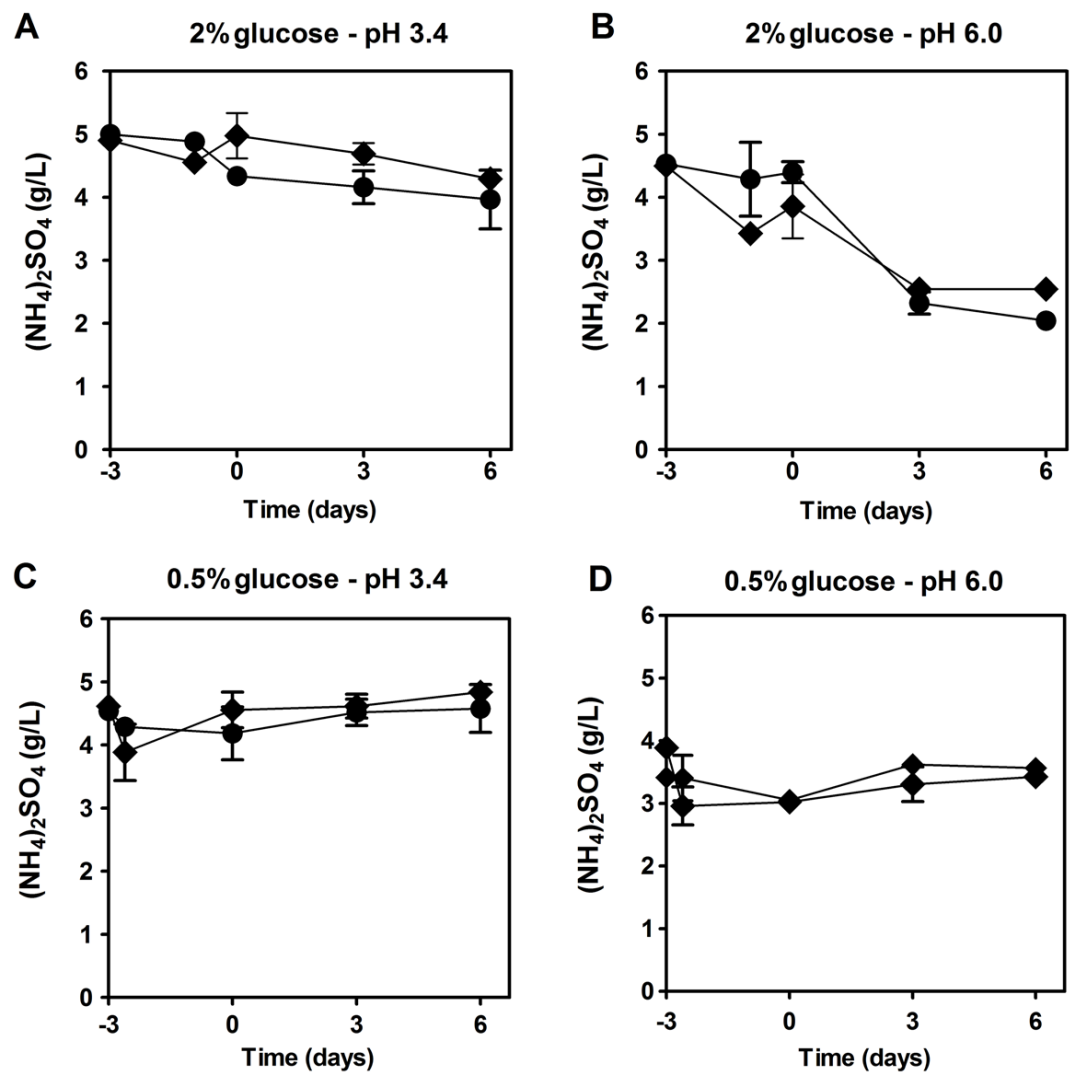
The majority of ammonium is not consumed remaining in the medium during culture aging Ammonium consumption (NH_4_)_2_SO_4_ by *S. cerevisiae* BY4742 cultured in SD media buffered to: pH 3.4 (A and C) or pH 6.0 (B and D), with 2% (A and B) or 0.5% (C and D) glucose and supplemented with: (◆) high and (●) low concentrations of auxotrophy-complementing amino acids. Day -3 represents the day of culture inoculation and day zero represents the beginning of aging experiments. Values are means ± SEM (n=3).

**Figure 3:**
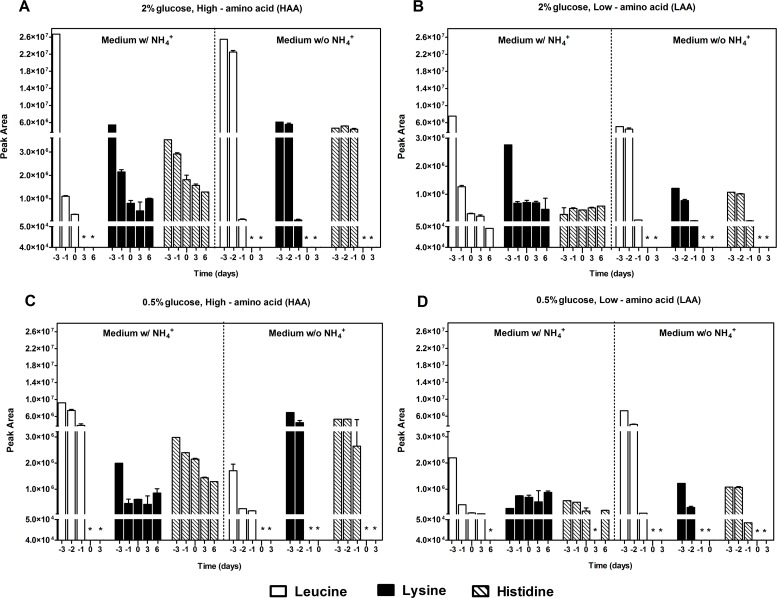
Ammonium,when present in the culture medium,impaired the consumption of theauxotrophy-complementing amino acids Leucine, lysine and histidine consumption of *S. cerevisiae* BY4742 cultured in SD media buffered to pH 3.4 with 2% (A and B) or 0.5% (C and D) glucose, supplemented with: high (A and C) and low (B and D) concentrations of auxotrophy-complementing amino acids (HAA and LAA, respectively), and with (w/) or without (w/o) NH_4_^+^ [0.5%, (NH_4_)_2_SO_4_]. Day -3 represents the day of culture inoculation and day zero represents the beginning of aging experiments. *(Peak values below detection limit ≈ 0). Values are means ± SEM (n=3).

To determine if the presence of NH_4_^+^ could be affecting cell viability by impairing the use of auxotrophy-complementing amino acids, we assessed the NH_4_^+^ and amino acid consumption during aging experiments in the culture medium. Regarding NH_4_^+^, there were no major differences for all the conditions tested (LAA and HAA media with 2% or 0.5% glucose at pH 3.4 and pH 6.0) and NH_4_^+^ was never completely consumed, remaining in the medium during culture aging (Fig. 2). Next, we measured by HPLC, under the same experimental conditions, the medium content of the auxotrophy-complementing amino acids in the presence and absence of NH_4_^+^. In HAA medium, either with 2% or 0.5% glucose, in the presence of NH_4_^+^ (Fig. 3A and 3C), from the three auxotrophic-complementing amino acids (leucine, lysine and histidine) only leucine was depleted from the medium after day 3 of the CLS experiment, at both pH values tested (Fig. 3A and 3C, and Fig. S2). On the contrary, in HAA medium without NH_4_^+^ either with 2% or 0.5% glucose, all three amino acids were completely depleted at both pH values, indicating that the presence of NH_4_^+^ seems to inhibit the complete consumption of these amino acids. In LAA medium pH 3.4, similar results were found, either with 2% or 0.5% glucose, in the presence or absence of NH_4_^+^ (Fig. 3B and 3D). However, in LAA medium in the presence of NH_4_^+^, poor amino acids consumption was noticed, particularly for leucine that was fully consumed for most conditions tested, except for LAA medium with NH_4_^+^ in which a fast loss of cell viability occurred (Fig. 3). At pH 6.0 similar results were obtained for the three amino acids, although a delay in their consumption was detected in comparison to pH 3.4 (Fig. S2 and Fig. 3).

### PKA and TOR pathways regulate the NH_4_^+^-induced CLS shortening during aging in culture medium

**Figure 4:**
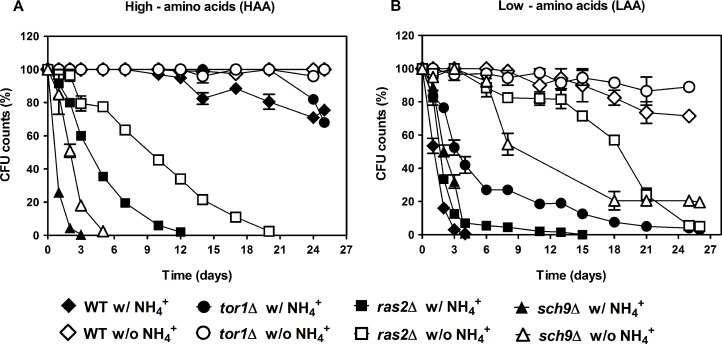
PKA and TOR pathways regulate the NH-induced CLS shortening during aging in culture medium Survival of wild-type *S. cerevisiae* BY4742, *tor1*Δ, *ras2*Δ and *sch9*Δ cells cultured in SD media buffered to pH 3.4 with 2% of glucose and supplemented with: high (A) and low (B) concentrations of auxotrophy-complementing amino acids (HAA and LAA, respectively), and with (dark symbols) or without (open symbols) NH_4_^+^ [0.5%, (NH_4_)_2_SO_4_] supplementation. In all the cultures, starting cell density was about 3.8 × 10^7^ cells/ml. Values are means ± SEM (n=3).

We have previously shown that for cells aged in water, that is under extreme caloric restriction, the toxic effects of NH_4_^+^ are mediated by activation of PKA and TOR and inhibition of SCH9 [31]. In order to unravel which nutrient signaling pathways might be involved in NH_4_^+^-induced CLS shortening in culture medium, where other nutrients might also interfere with these signaling pathways, *tor1*Δ, *ras2*Δ and *sch9*Δ cells were cultured in medium supplemented with low and high auxotrophic-complementing amino acids concentrations (LAA and HAA media), buffered to pH 3.4. The results showed that in HAA medium in the presence or absence of NH_4_^+^, both *ras2*Δ and *sch9*Δ cells loose viability rapidly in comparison to wild-type cells that do not present a significant loss of cell viability under these conditions (Fig. 4A). Hence, Ras2p and Sch9p seem to mediate survival in response to favorable conditions of amino acids (HAA medium) since their deletion resulted in a decrease of CLS in comparison to the wild type CLS (Fig. 4A) but do not seem to mediate the toxic response to NH_4_^+^ as in their absence the CLS of both deleted strains decreased slower than in medium with NH_4_^+^. On the contrary, in LAA medium with NH_4_^+^, but not its absence, Ras2p and Sch9p appear to mediate cell death, an extension of CLS being observed upon their deletion in comparison to the wild-type cells (Fig. 4B). As verified in the previous section for the wild type cells, the absence of NH_4_^+^ in LAA medium also significantly decreased the death phenotype induced by NH_4_^+^ in these mutants, without however, presenting a complete abolishment as observed for wild type cells (Fig. 4B). These results suggest that in amino acid restriction conditions without NH_4_^+^, Sch9p and Ras2p are necessary for cell survival. Tor1p does not specifically respond to amino acid concentrations in medium, displaying an extended CLS in both LAA and HAA medium (Fig. 4A and 4B). However, Tor1p may be involved in mediating NH_4_^+^-induced cell death in low amino acid concentrations, presenting an extension of CLS upon its deletion.

### The negative effect of ammonium during aging is associated with replicative stress induction

In order to further clarify the negative effects of NH_4_^+^ during aging, and since signaling though RAS/PKA pathway may inhibit Rim15p and consequently compromise entrance in G0 state [33], we also assessed whether NH_4_^+^ could be inducing replicative stress by monitoring cell cycle progression by flow cytometry along aging experiments under the different conditions tested above.

**Figure 5:**
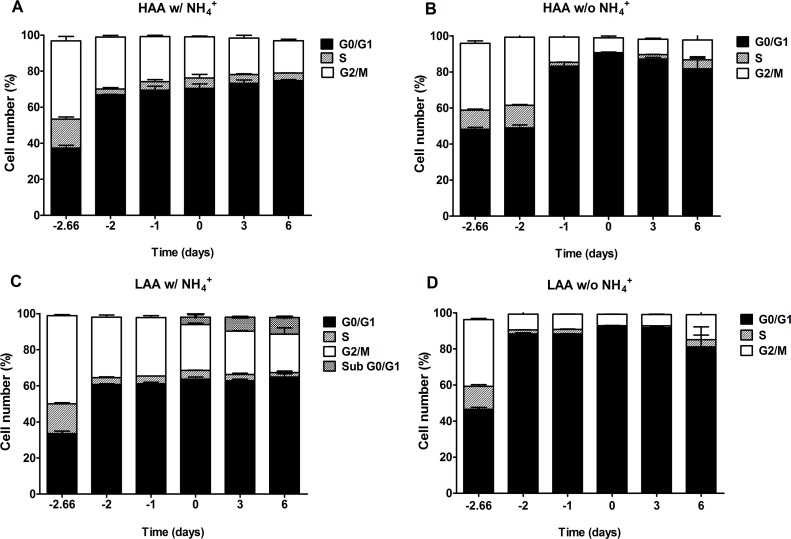
The negative effect of ammonium during aging is associated with replicative stress induction Cell cycle analysis of *S. cerevisiae* BY4742 cells cultured in SD media buffered to pH 3.4 with 2% glucose and supplemented with: high (A and B) and low (C and D) concentrations of auxotrophy-complementing amino acids (HAA and LAA, respectively) and with (w/) (A and C) or without (w/o) (B and D) NH_4_^+^ [05%, (NH_4_)_2_SO_4_] supplementation. Day -3 represents the day of culture inoculation and day zero represents the beginning of aging experiments. Values are means ± SEM (n=3).

The results from the cell cycle analysis demonstrate that the percentage of cells in G0/G1 at stationary phase was lower in the presence of NH_4_^+^ for cells cultured both in medium with high or low amino acid concentrations with 2% glucose, this value being particularly low for LAA medium with NH_4_^+^, therefore indicating that the presence of NH_4_^+^ inhibits a proper cell cycle arrest (Fig. 5A and 5C). In media lacking NH_4_^+^ (Fig. 5B and 5D), either LAA or HAA, the percentage of cells in G0/G1 at stationary phase was close to 90% indicating a proper arrest of the culture.

**Figure 6:**
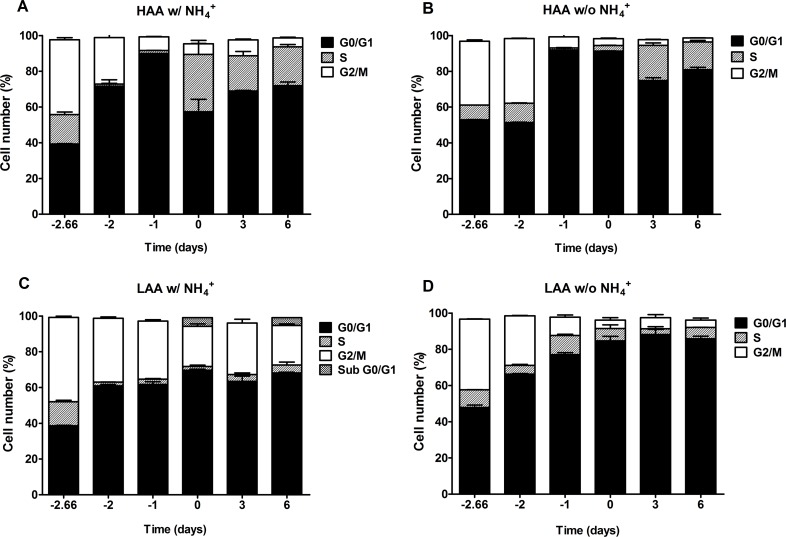
In medium buffered to pH 3.4, ammonium inhibits a proper cell cycle arrest under caloric restriction only for cells cultured with low amino acid concentrations Cell cycle analysis of *S. cerevisiae* BY4742 cells cultured in SD media buffered to pH 3.4 with 0.5% glucose and supplemented with: high (A and B) and low (C and D) concentrations of auxotrophy-complementing amino acids (HAA and LAA, respectively) and with (w/) (A and C) or without (w/o) (B and D) NH_4_^+^ [0.5%, (NH_4_)_2_SO_4_] supplementation. Day -3 represents the day of culture inoculation and day zero represents the beginning of aging experiments. Values are means ± SEM (n=3).

**Figure 7:**
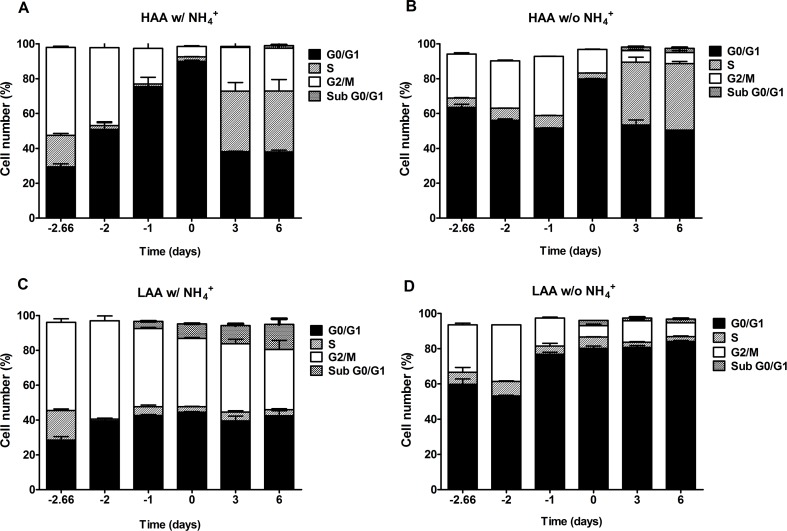
In medium buffered to pH 6.0, ammonium inhibits a proper cell cycle arrest under caloric restriction only for cells cultured with low amino acid concentrations Cell cycle analysis of *S. cerevisiae* BY4742 cells cultured in SD media buffered to pH 6.0 with 0.5% glucose and supplemented with: high (A and B) and low (C and D) concentrations of auxotrophy-complementing amino acids (HAA and LAA, respectively) and with (w/) (A and C) or without (w/o) (B and D) NH_4_^+^ [0.5%, (NH_4_)_2_SO_4_] supplementation. Day -3 represents the day of culture inoculation and day zero represents the beginning of aging experiments. Values are means ± SEM (n=3).

In cells cultured under calorie restriction conditions, the presence of NH_4_^+^ inhibits a proper cell cycle arrest only for cells cultured in medium with low amino acid concentrations supplemented with NH_4_^+^, in medium buffered to pH 3.4 or pH 6.0 (Fig. 6C and 7C). In medium lacking NH_4_^+^ or with high amino acid concentrations and NH_4_^+^ (Fig. 6A, 6B and 6D; Fig. 7A, 7B and 7D) cells presented a proper arrest in G0/G1 phase. These results show that there is a strong correlation between faster loss of cell viability during aging and the inability of the culture to enter G0/G1 and thus suggest the occurrence of a replicative stress induced by NH_4_^+^.

## DISCUSSION

The budding yeast *S. cerevisiae* is nowadays one of the most exploited models to study the environmental and genetic factors affecting longevity. Using this model we have previously shown that manipulation of NH_4_^+^ concentration in the culture and/or aging medium can drastically affect chronological lifespan (CLS), especially in amino acid restricted cells [31, 32]. In other reports methionine restriction is described as prolonging lifespan in yeast and mammalian cells [34, 35], recently, being demonstrated that the difference in CLS extension presented by EUROSCARF wild-type long-lived BY4741 strain and the short-lived BY4742 is due to the capacity of biosynthesis of methionine presented by the latter strain [36]. The results presented herewith provide evidence that culturing *S. cerevisiae* in amino acid restriction conditions, in the presence of a non-limiting good nitrogen source (NH_4_^+^ and glutamine, in the present work) greatly reduces CLS in association with replicative stress induction. The CLS shortening is mainly due to the abundance of the non-limiting good nitrogen source in the culture medium, since in its absence no significant difference in CLS was observed between cells cultured with or without amino acid restriction (LAA or HAA media).

Evidence from several studies increasingly suggests that not only caloric restriction but a balance of different nutrients have a pivotal role in regulating lifespan [37]. In fruit flies DR-induced longevity was achieved by reducing calories of the source of protein, yeast extract, which provided greater lifespan extension than isocaloric reduction of sucrose [38]. Similarly, addition of essential amino acids to dietary restriction in sucrose plus yeast-based diet diminishes longevity extension [39]. In mice, a current study establishes a ratio between carbohydrate and protein that significantly affects longevity without being influenced by total calorie intake, demonstrating that increasing of the protein: carbohydrate ratio leads to mTOR activation in association with low glucose and high levels of circulating branched-chain amino acids [40]. In our present work, the caloric restriction effect could be observed under amino acid restriction conditions, but only in the presence of NH_4_^+^, not in its absence. Furthermore, in LAA medium under calorie restriction conditions, a much faster decrease in cell viability is observed in the presence of NH_4_^+^ than in its absence, indicating that NH_4_^+^ *per si*, independently of glucose concentration in the medium during aging, is a major accountable for the CLS shortening observed under amino acid restriction.

As concerns the influence of pH in aging, our results show that buffering media not only to pH 6.0 (Fig. 1B) but also to pH 3.4 (Fig. 1A) largely extends CLS in HAA media with or without NH_4_^+^ and in LAA media without NH_4_^+^ (Fig. S1 and Fig. 1A). Buffering LAA media in the presence of NH_4_^+^ to either pH 3.4 or pH 6.0 had no measurable effect on CLS (Fig. 1A and 1B). This is in agreement with our previous work where we showed that pH (between 2.8 and 7.0) did not have a significant effect on NH_4_^+^-induced CLS shortening of cells aged in water. In another study it was also shown that buffering of aging cultures to pH 6.0 is sufficient to increase CLS as compared to that measured at pH 3.0, an effect that was attributed to the dissociation of acetic acid, resulting in a decrease of the acetic acid concentration in the undissociated form with increasing pH (the anion acetate is prevalent at pH 6.0) [18]. Interestingly, our results demonstrate that buffering media to a low pH such as 3.4, where no significant differences in the acetic acid protonated form are encountered as compared to the unbuffered medium (medium pH after 96 hours in 2% glucose SC medium is around 2.87 [18]), considerably extends CLS suggesting that in our conditions acetic acid does not seem to be involved in the CLS shortening.

A recent study demonstrated that *S. cerevisiae* sequentially uses amino acids and NH_4_^+^, with no marked differences in the order of assimilation of the nitrogen compounds between strains tested, and that this sequential use is largely determined by both the kinetic characteristics and the regulation of the transporters of amino acids and NH_4_^+^. The initial concentrations of these compounds did not alter the order in which they were consumed, except for arginine and NH_4_^+^ [41].

In our data, the majority of NH_4_^+^ was not consumed in the two conditions tested (LAA and HAA) and its presence in the medium was associated with a decrease of the consumption of the essential amino acids. This effect of NH_4_^+^ on the consumption of the essential amino acids is more accentuated in LAA medium, particularly for leucine which was associated with a drastic decrease of CLS exhibited in this condition. The NH_4_^+^ concentration in the culture medium has been described to influence NH_4_^+^ consumption patterns [41]. In our studies, the variances encountered regarding nitrogen consumption could be due to the NH_4_^+^ concentration [0.5%, (NH_4_)_2_SO_4_], the common concentration present in laboratory culture media, as well as to the use of auxotrophic strains that could present alterations in nitrogen regulation.

About the involvement of nutrient signaling pathways in CLS regulation in the conditions tested, our results suggest that Ras2p and Sch9p seem to mediate survival in response to favorable conditions of amino acids (HAA medium) since its deletion resulted in a decrease of CLS in comparison to the wild type CLS (Fig. 4A), but they appear not to be involved on the toxic response to NH_4_^+^ since the CLS of both deleted strains decreased slower in the HAA medium lacking NH_4_^+^ than in its presence. On contrary, in LAA medium with NH_4_^+^, Ras2p and Sch9p seem to mediate cell death, with the deficiency on these proteins resulting in an extension of CLS in comparison to the wild-type cells. These results may be correlated to other studies where NH_4_^+^ in the presence of glucose triggers a rapid PKA activation via Sch9p leading to fermentative growth and inhibiting a stress resistance response [42, 43]. Interestingly, in LAA medium without NH_4_^+^ these proteins adopt a pro- survival role as demonstrated by the decrease in CLS extension in comparison to wild type cells upon their deletion. Both Ras2p and Sch9p have been involved in longevity regulation with deletions of these genes extending CLS. In our results, the two deleted strains were overall less long-lived than the WT strain, in agreement with other works where medium composition and strain background influence longevity of these deleted strains [14, 44]. In fact, Sch9p has been described as being more sensitive to nutrients, including to NH_4_^+^, than other deleted strains tested in medium with YNB and amino acids variations, which had major influence on the CLS of the *sch9*Δ strain [14, 31].

Additionally, Tor1p seems to mediate cell death in low amino acid concentrations, both in the presence and absence of NH_4_^+^. Amino acid sensing can occur through two conserved signal transduction pathways, the eIF2*α* (eukaryotic initiation factor 2*α*) kinase GCN2 (general amino acid control non-derepressible 2) and TOR kinase [29]. In yeast, the GCN2-based GAAC response is activated when individual amino acids are deficient, on the other hand the presence of certain amino acids, such as leucine, activates TORC1, increasing TORC1-dependent phosphorylation of Sch9p [45, 46]. In this way, the observed inhibition of total leucine depletion in the presence of NH_4_^+^ in LAA medium (Fig. 3B) could lead to an activation of the TOR pathway and to a rapid decrease of the CLS. In contrast, the depletion from the medium of an individual amino acid such as leucine, exhibited in the other conditions tested (Fig. 3), could activate a GCN2-based GAAC response, resulting in the observed extended longevity (Fig. 1). A recent work in mammalian cells has also demonstrated a parallel activation of the GAAC pathway and mTOR deactivation in response to serum/glutamine starvation. mTOR reactivation was dependent on exogenous leucine and leucine transporter up-regulation [47]. Our data showing interplay between NH_4_^+^ and amino acids during yeast chronological aging in the culture medium further suggest the GCN2 role in longevity as a direct amino acid starvation response activator. Further studies in yeast, to unravel ammonium's amino acid consumption inhibition and a possible role of the amino acid sensing kinases GCN2 and TOR1, could now be explored as a model to more complex systems.

A correlation between GCN2 and autophagy has also been described for amino acid starvation conditions where only auxotrophy-complementing amino acids are capable of inducing an autophagic trafficking pathway completely dependent on Gcn2p and Gcn4p, and distinct from macroautophagy induced by total nitrogen starvation [48]. Our findings suggest that the presence of a non-limiting rich nitrogen source, like NH_4_^+^, in LAA medium and the associated impairment of essential amino acids consumption could be related with an inhibition of macroautophagy and promotion of a lower autophagic response induced by amino acid starvation which may lead to a rapid loss of cell viability. This hypothesis is supported by our previous results showing that autophagy was not induced in a 24 hour amino acid-starvation conditions *versus* a 24 hour nitrogen-starvation condition [31].

The observed negative effect of NH_4_^+^ during aging appeared to be associated with replication stress induction. Actually the results from the cell cycle analysis indicate that the presence of NH_4_^+^ inhibits a proper cell cycle arrest for cells cultured both in medium with high or low amino acid concentrations with 2% glucose (Fig. 5A and 5C) as compared with a proper arrest of the culture in the same media lacking NH_4_^+^. In cells cultured under calorie restriction conditions, the presence of NH_4_^+^ also inhibits a proper cell cycle arrest but only for cells cultured in LAA medium. These results are in agreement with previous evidences showing that cells with auxotrophys fail to coordinate proper cell cycle arrest upon starvation for the respective auxotrophy while cells starved for natural nutrients such as glucose, phosphate or sulfate achieve a prompt cell cycle arrest [16, 25]. Our results now show that the failure of auxotrophy-complementing amino acid limited cells to enter proper cell cycle arrest is due to the presence of NH_4_^+^ in the culture medium (and possible any other rich nitrogen source). Also, the observed proper cell cycle arrest for cells under caloric restriction in medium with high amino acid concentrations and NH_4_^+^ (Fig. 7A) strongly suggests that glucose as the limiting nutrient is the main responsible for signaling entrance in G0 phase. This is in line with previous results showing that yeast viability and failure to commit to the cell division cycle depends on which nutrient is limited first, based on a survival strategy in the face of inadequate nutrition [16, 49].

In summary, our results indicate that NH_4_^+^ promotes aging through the regulation of the same pathways as the ones involved in glucose-induced aging and further substantiate that the activation of these pathways is cumulative. Consequently, excess of both nutrients highly contribute to aging and decrease of only one of them will alleviate signaling and promote longevity. However, decreasing nutrient concentration in the medium is only beneficial if the limiting nutrient can induce cells to properly enter a G0 phase on its exhaustion. Furthermore, the CLS shortening induced under auxotrophy-complementing amino acids restriction appeared to be mainly due to the presence of a non-limiting rich nitrogen source in the culture medium (NH_4_^+^ and glutamine, in the present work) and that the abundance of NH_4_^+^ in the medium could be affecting cell viability by impairing the use of the essential amino acids. Moreover, the beneficial effect of nitrogen restriction on CLS can only be observed for NH_4_^+^ or non-essential amino acids like glutamine, but not for the essential amino acids of the strain BY4742 used in the present work. The effect of rich nitrogen source restriction seems to be particularly relevant if there is simultaneous restriction of an essential amino acid. Thus, considering the use of rich nitrogen sources, the reduction in its abundance in the medium rather than the nitrogen source specificity appears to be more important for the extension of life span.

As a final remark, herewith we present for the first time evidence supporting that NH_4_^+^ is a key determinant in the nutritional balance required for the beneficial effect of dietary restriction on longevity, therefore shedding light on how nutrient balance of dietary regimes could also affect longevity in higher eukaryotes.

## METHODS

### Strains and growth conditions

*Saccharomyces cerevisiae* strain BY4742 (*MAT*a *his3Δ*1 *leu2Δ*0 *lys2Δ*0 *ura3Δ*0) (EUROSCARF, Frankfurt, Germany) and the respective knockouts in *RAS2*, *SCH9*, and *TOR1* genes, were used. For experiments with stationary phase cells with or without restriction of auxotrophy-complementing amino acids (essential amino acids), cells were cultured at 26 ºC, 150 rpm, until stationary phase was reached, in defined minimal medium (SD medium) containing 0.17% yeast nitrogen base without amino acids and without ammonium sulphate (Difco, BD), 2% or 0.5% D-glucose; supplemented with or without ammonium sulphate (5 g/L) or glutamine (700 mg/L), and with low (10 mg/L histidine, 10 mg/L lysine, 60 mg/L leucine and 100 mg/L uracil) or high (50 mg/L histidine, 50 mg/L lysine, 300 mg/L leucine and 100 mg/L uracil) concentrations of essential amino acids. Citrate phosphate was used for buffering medium to pH 3.4 (28.2 mM Na_2_HPO_4_ and 35.9 mM citric acid) or to pH 6.0 (64.2 mM Na_2_HPO_4_ and 17.9 mM citric acid). At stationary phase, cell density was adjust to about 3.8 × 10 ^7^cells/ml. Viability of stationary 3 day old cultures was considered to be 100% of survival and this was considered day 0 of the aging experiment. Cell viability was assessed by Colony Forming Units (CFU) at day 0 (100% viability) and in subsequent days, as indicated, of culture aliquots incubated for 2 days at 30 °C on YEPD agar plates.

### Ammonium and glucose determination

Ammonium and glucose in the culture media were quantified at the indicated time points using an ammonia assay kit (Sigma) and a glucose oxidase (GOD) assay (Roche Diagnostics GmbH), respectively and following the manufacturer's instructions.

### Cell cycle analysis

To measure DNA content, cells were stained with SYBR Green I as previously described [50] and staining was assessed by flow cytometry. Flow cytometry analysis of the experiments was performed in a BD™ LSR II flow cytometer and thirty thousand cells per sample were analyzed. Offline data was analyzed with the flow cytometry analysis software package FlowJo 7.6.1.

### HPLC quantification of amino acids

Amino acids in the media were assayed by reverse phase high-performance liquid chromatography, using a UV/VIS-155 Gilson detector at 338 nm and a HICHROM 5 C18 (5μm 25cm x 4.6 mm) column at 40° C, as previously described [51] with minor changes. The amino acid derivatization reagent was prepared fresh each day by dissolving 25 mg of OPA (*o*-phthaldialdehyde, Sigma) in 1.0 mL of methanol and 0.15 mL of 1 M potassium tetraborate buffer (pH 9.5), and 26 μL 2-mercaptoethanol was added. This derivatization reagent was further diluted with 5 mL of 1 M potassium tetraborate buffer (pH 9.5) to obtain the final working solution. The derivatization of the amino acids in the samples was performed in an automated fashion using a Gilson 234 auto-injector. Mobile phases were (A) aqueous solution of 175 mM Na_2_H_2_PO_4_ and 125 mM propionic acid, HPLC grade Acetonitrile and HPLC grade water at pH 7.8 (40:8:52 by vol.) and (B) HPLC grade acetonitrile, HPLC grade methanol and HPLC grade water (30:30:40 by vol.)

### Statistical analysis

Values presented in graphs represent means and standard deviations from three independent experiments (± SEM n=3). Statistical analyses were performed by two-way ANOVA. P < 0.05 was considered statistically significant.

## SUPPLEMENTARY MATERIAL, FIGURES


