# Supplementary material for: Ammonium is a key determinant on the dietary restriction of yeast chronological aging in culture medium
Source: Oncotarget. 2014 Dec 3;6(9):6511–23. doi: 10.18632/oncotarget.2989 (PMC4466630; doi:10.18632/oncotarget.2989)
Supplement: Supplementary file 1 [file oncotarget-06-6511-s001.pdf]

Ammonium is a key determinant on the dietary restriction of yeast chronological aging in culture medium

Supplemental Material

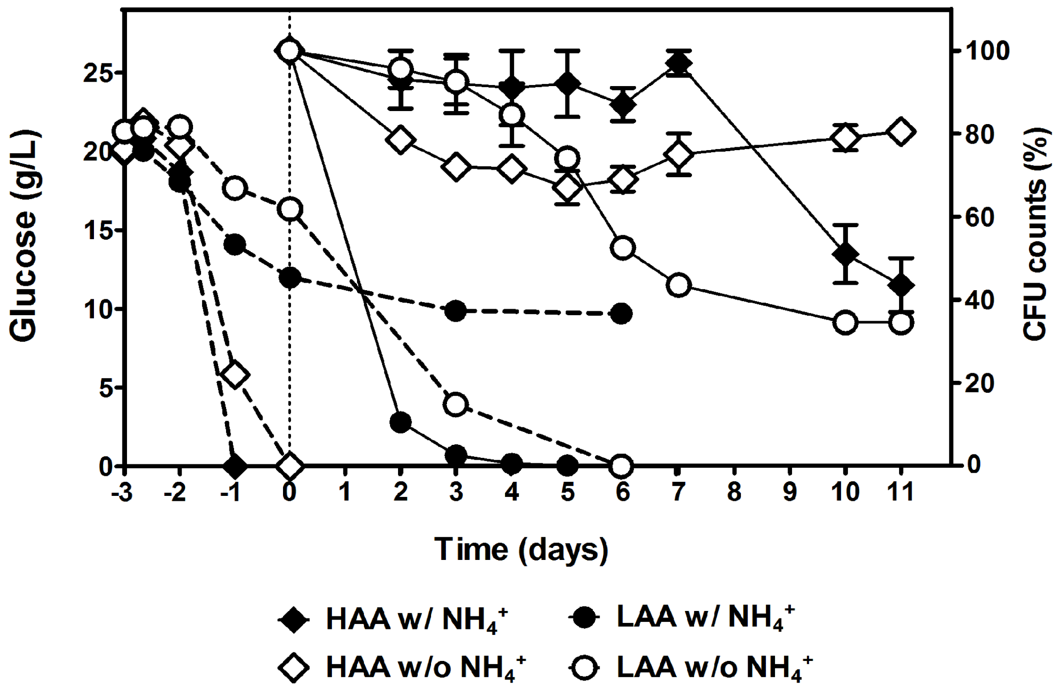

Figure S1: CLS shortening under amino acid restriction is reverted by removing the ammonium from culture medium (non-buffered SD medium). Glucose consumption and survival of wild-type *S. cerevisiae* BY4742 cultured in SD media with 2% and supplemented with: high and low concentrations of auxotrophy-complementing amino acids (HAA and LAA, respectively), and with (w/) or without (w/o) NH_4^+ [0.5%, $(NH_4)_2SO_4$]. In all the cultures, starting cell density was about 3.8 x 10^7 cells/ml. Day -3 represents the day of culture inoculation and day zero represents the beginning of aging experiments. Values are means ± SEM (n=3)

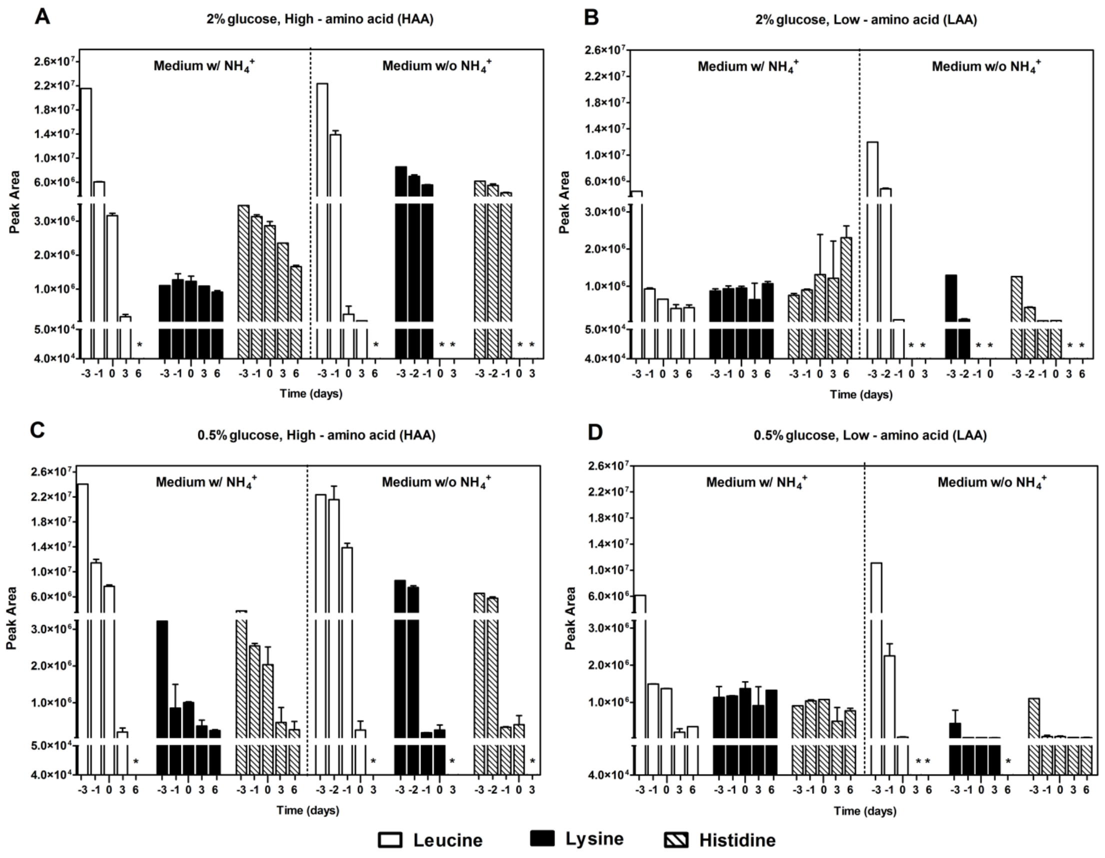

Figure S2: **Ammonium, when present in the culture medium, impaired the consumption of the auxotrophy-complementing amino acids, in media buffered to pH 6.0.** Leucine, lysine and histidine consumption by *S. cerevisiae* BY4742 cultured in SD media buffered to pH 6.0 with 2**%** (A and B) or 0.5% (C and D) glucose, supplemented with: high (A and C) and low (B and D) concentrations of auxotrophy-complementing amino acids (HAA and LAA, respectively), and with (w/) or without (w/o) NH_4^+ [0.5%, $(NH_4)_2SO_4$]. Day -3 represents the day of culture inoculation and day zero represents the beginning of aging experiments. *(Peak values below detection limit ≈ 0). Values are means ± SEM (n=3)
